# A Rare Variant of Zinner Syndrome Involving Ectopic Ureteral Implantation into the Seminal Vesicle Causing Recurrent Epididymitis

**DOI:** 10.1155/2024/9432939

**Published:** 2024-03-18

**Authors:** Michael Zaliznyak, Aaron Baer, Joshua Trierweiler, Thomas Landon, Zachary Hamilton

**Affiliations:** ^1^Saint Louis University School of Medicine, St. Louis, MO, USA; ^2^Department of Urology, Saint Louis University School of Medicine, St. Louis, MO, USA

## Abstract

Zinner syndrome is a rare congenital anomaly characterized by a triad of renal dysgenesis/agenesis, cysts in the ipsilateral seminal vesicle, and ejaculatory duct obstruction. Though often diagnosed in infancy, the diagnoses can be incidentally found in adults who present with nonspecific genitourinary symptoms including dysuria, ejaculatory dysfunction, or genital pain. We present an unusual case of a 29-year-old male patient who presented to the emergency department with recurrent testicular pain and hematospermia and was found to have an atrophic right kidney with an ectopic ureter implanting into a cystic seminal vesicle. These findings were consistent with a rare subvariant of Zinner syndrome only previously described four times in the literature. We performed a robotic-assisted laparoscopic ectopic nephroureterectomy with sparing of his seminal vesicle. To our knowledge, this is the first report to describe the safe and effective use of robotic surgery in this setting to remove affected anatomy while preserving the patient's seminal vesicle.

## 1. Introduction

Ectopic insertion of the ureter is a rare congenital anomaly occurring in 0.05-0.025% of men [1]. The condition has been associated with Zinner syndrome, a rare triad of renal dysgenesis/agenesis, cysts in the ipsilateral seminal vesicle, and ejaculatory duct obstruction, that has been described in approximately 100 cases [2]. Congenital renal collecting system abnormalities are commonly diagnosed in infancy, but some patients may go undiagnosed into adulthood when they are discovered incidentally during workup for recurrent genitourinary complaints [3, 4]. Although asymptomatic patients are often managed conservatively, if significant symptoms are present, then surgical intervention becomes necessary [5].

We present an unusual case of an adult found to have an atrophic right kidney with ectopic ureteral insertion into the ipsilateral seminal vesicle, who was successfully treated with robotic-assisted laparoscopic ectopic nephroureterectomy with sparing of the affected seminal vesicle. The positioning of the ureter within the seminal vesicle represents a rare subvariant of Zinner syndrome, which to our knowledge has only been previously described four times in the literature [3, 6–8].

## 2. Case Presentation

### 2.1. Brief History

**Figure 1:**
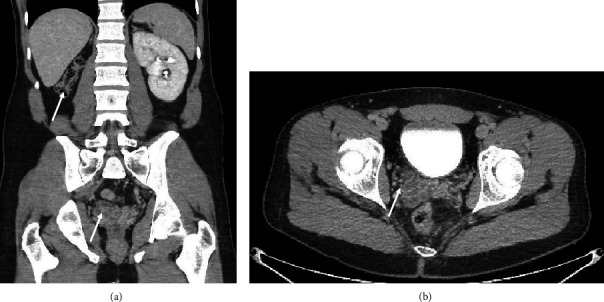
Abdominopelvic computerized tomography (CT). (a, b) Coronal and axial images reveal atrophic right kidney with cystic right seminal vesicle.

**Figure 2:**
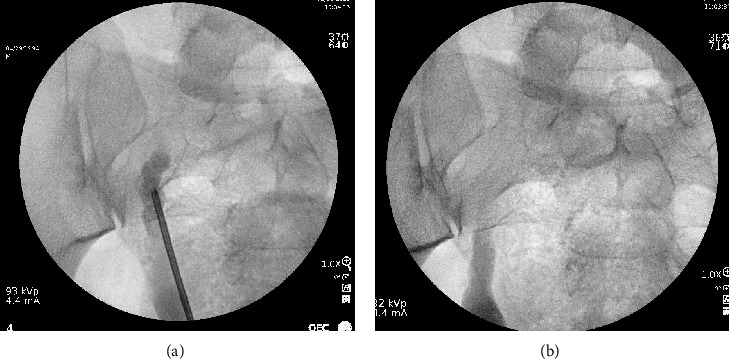
Retrograde ureterogram imaging showing (a) a rigid ureteroscope in the right seminal vesicle that is filled with contrast and (b) a dilated distal right ureter coming off the seminal vesicle.

The patient was a 29-year-old Caucasian male who presented to the emergency department (ED) with right-sided testicular discomfort, testicular swelling, dysuria, and hematospermia. Urinalysis was obtained and showed 3+ leukocyte esterase, and urine culture was positive for *Escherichia coli*. His symptoms resolved with antibiotics, and he was discharged. Subsequently, he presented a second time to the ED within one month for similar symptoms of dysuria and right-sided testicular discomfort and swelling. Computed tomography (CT) urogram obtained at that time revealed a normal left-sided collecting system and a congenitally atrophic right kidney with ipsilateral ectopic ureter implanting into a dilated cystic seminal vesicle (Figure 1). The right seminal vesicle was measured as 3.3 × 2.5 × 4.6 cm and contained approximately 10-15 cysts. His right ectopic ureter was dilated distally to the L5-S1 level. No hydronephrosis was noted. The patient was unaware of this congenital finding prior to his CT results. There were no prenatal imaging findings included in his electronic medical record. He was subsequently referred to an outside urologist who took the patient to the operating room and performed a cystoscopy. During the cystoscopy, the right ureteral orifice was observed in the prostate fossa just distal to the verumontanum. A cone-tip catheter was placed into this orifice, and a gentle retrograde ureterogram was performed, showing a dilated seminal vesicle. At this point, a ureteroscopy was performed and confirmed the insertion of the right ureter into the ipsilateral seminal vesicle. A retrograde pyelogram (Figure 2) was then performed showing a notably dilated right ureter. The patient was then referred to our care for further evaluation and possible surgical intervention.

### 2.2. Diagnosis

On presentation, he was again reporting continued right-sided scrotal pain and hematospermia. His family and social history were noncontributory. He had no significant urologic past medical history to this point. We performed a right-sided seminal vesiculoscopy via the ejaculatory duct and performed a repeat retrograde pyelogram visualizing the filling of the right seminal vesicle with contrast from the ectopic right ureter, confirming the diagnosis. The patient was counselled that his recurrent symptoms were likely secondary to his atrophic right kidney with ectopic ureteral implantation into his right seminal vesicle. Given that he was continuing to have hematospermia and right-sided episodes of epididymitis, we discussed the removal of his ectopic ureter and his atrophic right kidney. On review of his CT imaging, his seminal vesicle cystic burden was determined to be mild. Given this, we discussed the possibility of sparing the seminal vesicle. The patient was concerned that removing the seminal vesicle may result in reduced ejaculation and erectile dysfunction. The patient was cautioned that preserving the seminal vesicle may result in incomplete resolution of his symptoms. He elected to undergo a right nephroureterectomy, and a robotic approach was selected. Informed consent was obtained. The patient and his partner declined fertility testing at this time.

### 2.3. Intervention

**Figure 3:**
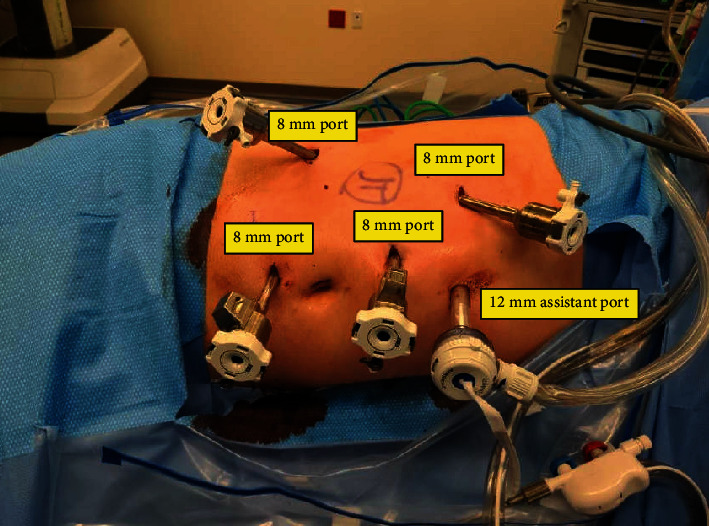
Port placements for robotic-assisted nephroureterectomy.

**Figure 4:**
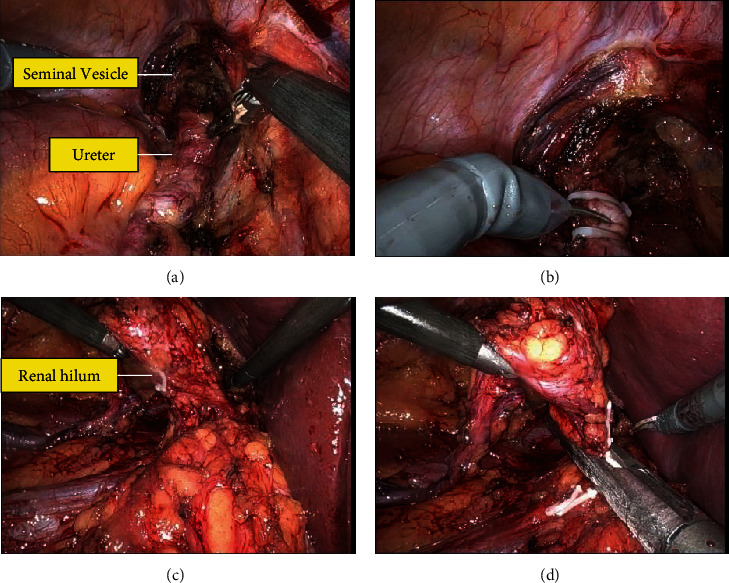
The ectopic ureter was identified implanting into the seminal vesicle (a). The ureter was transected at the level of its insertion using Hem-o-lok clips (b). The renal hilum was then identified (c). The atrophic kidney was identified, and a complete nephrectomy was performed (d).

**Figure 5:**
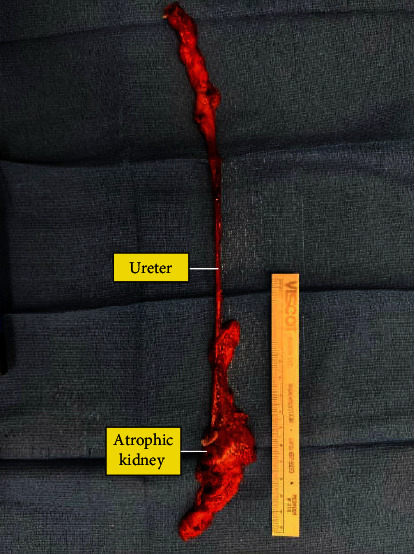
Gross specimen of the right atrophic kidney and ureter.

After anesthesia was induced, the patient was placed into the left lateral decubitus position with the right flank exposed. His right flank was prepped and draped in a sterile fashion. We placed 4 robotic arms in a modified diamond configuration with a 10/12 AirSeal assist superior to the umbilicus and the robot was docked (Figure 3). The right colon was reflected medially. We identified the ureter as it was crossing over the iliac. The ureter had a normal appearance cephalad to the common iliac but was dilated caudad to this area. We circumferentially dissected out the ureter distally down towards the level of the seminal vesicle. We identified the ureter inserted into the seminal vesicle (Figure 4(a)). We were able to dissect the ureter to its insertion and then transected the ectopic ureter at the level of the seminal vesicle using Hem-o-Lok clips (Figure 4(b)). Thus, the seminal vesicle was left intact. We minimized cautery use around the tip to avoid damage to the seminal vesicle over the neurovascular bundle. Next, we traced up the ureter cephalad. We were able to dissect it towards the level of the atrophic right kidney. Next, we continued to reflect the right hemicolon medially, and we were able to identify the adrenal gland. This was spared and kept in a superior direction. Thus, by dissecting around the adrenal gland in the lower pole portion, we were able to identify the area of the renal hilum (Figure 4(c)). We did identify 2 renal veins. One lower pole renal vein was clipped with Hem-o-Lok clips. The second main renal vein with the right renal artery was transected en bloc with a 45 mm endovascular staple fire. At this point in time, the kidney was freely detached and we had thus performed a complete nephrectomy with total ureterectomy (Figure 4(d)). The robot was undocked. The patient tolerated the procedure and was returned to post-op anesthesia holding in stable condition. The final pathology showed a benign ureter and atrophic kidney (Figure 5).

The patient was discharged on postoperative day 1. On postoperative day 5, the patient returned to the ED with urinary retention for 2 hours and left-sided testicular pain. He denied any right-sided testicular pain. Urine culture was positive for E. coli, and he was prescribed antibiotics and discharged. At his first two-week postoperative clinic visit, he endorsed the complete resolution of symptoms. The urine culture obtained at this visit was clean. The patient was instructed to contact the clinic if he experienced any new or persistent symptoms. At the time of this report, he is now 6 months out from surgery and has not reported any new or persistent symptoms. He has had no additional ED admissions.

## 3. Discussion

Zinner syndrome is considered the male equivalent of the female Mayer-Rokitansky-Kuster-Hauser syndrome. It is a rare disorder, estimated to occur in 0.0046% of the population [9]. It is associated with the abnormal development of the mesonephric (Wolffian) duct in the first trimester of gestation. An insult of the distal part of the mesonephric duct leads to atresia of the ejaculatory duct and abnormal ureteral budding. Incomplete migration of the ureteric bud and failure to connect with the metanephros result in renal agenesis [10]. However, the seminal vesicles continue to develop with insufficient drainage due to the obstruction of the ejaculatory duct, resulting in distention and cyst formation [11]. Our case is unusual in that it represents a rare variant of Zinner syndrome in which the patient is noted to have an ectopic ureter implanting into the cystic seminal vesicle. To our knowledge, this is only the 5th such case described in the literature [3, 6–8].

Congenital anatomic abnormalities of the genitourinary tract are rarely diagnosed in adulthood. Although rare, they may become discovered during the workup of recurrent adult pathologies. The patient described in our case presented with recurrent epididymitis, testicular discomfort, and hematospermia, which are among the most frequently presenting symptoms. Our case suggests that providers should maintain a high level of suspicion when treating patients with recurrent genitourinary complaints and should consider prompt workup with imaging studies to assess for potentially correctable abnormal anatomy.

There are no agreed-upon guidelines for routine screening of conditions related to Zinner syndrome. Although overall rare, the incidence of Zinner syndrome in patients with congenital solitary kidneys is approximately 10-12%, making it reasonable to routinely screen patients in this setting [12]. In a large, single-center study of patients with congenital solitary kidneys, the authors recommended routine pelvic ultrasound between 1 and 3 months of age and at every year of age divisible by 5, as well as annual follow-ups to review medical history, measure serum creatinine, obtain a urinalysis for proteinuria, and to perform a physical examination [12]. Lower urinary tract symptoms related to Zinner syndrome are thought to be directly associated with cystic accumulation and progressive cystic growth, and prompt diagnoses and treatment of cystic burden showed good prognosis in 75% of cases [12].

An important consideration in treating patients with seminal vesicle cysts is the preservation of fertility. Patient age and desire for future fertility must be carefully weighed during preoperative evaluation. Patients with Zinner syndrome often have abnormal semen parameters, with measured ejaculate volume and total sperm count down to 50% of reference ranges [13]. Preservation of the seminal vesicle in the treatment of Zinner syndrome may offer a patient benefit towards future fertility. Additionally, seminal vesicle sparing may reduce the risk of neurovascular injury and improve erectile functional outcomes following surgery. However, the cysts on the seminal vesicles are thought to be the cause of the common presenting physical symptoms of Zinner syndrome, including dysuria, and scrotal or perineal pain. Thus, the decision to preserve the seminal vesicle must be weighed against the risk of incomplete symptom resolution. Further, preservation of the seminal vesicles is not always possible in complex settings such as in patients with anatomical malformations or in patients with significant cystic burden. A conservative approach involving organ-sparing cyst removal has been described [14]; however, any procedures that spare the seminal vesicle do carry a risk of symptom reoccurrence. Although fertility preservation has been described with the seminal vesicle-sparing treatment of Zinner syndrome, large cohort studies and long-term data are limited given the sparsity of cases.

Minimally invasive robotic surgery has been shown to be safe and effective in the management of patients with symptomatic variants of Zinner syndrome. However, when pathology involves the seminal vesicle, such as in our case, patients must be counselled on the risk of infertility as a result of surgery. Only two of the four previously described cases of ectopic ureteral implantation into a seminal vesicle were treated surgically, and the seminal vesicle in each was excised [3, 8]. In one additional similar case of Zinner syndrome which involved an ectopic ureter originating from a supernumerary kidney, the seminal vesicle was also unable to be spared [15]. In our case, we were able to preserve the affected seminal vesicle by excising the ureter at its base. The decision to preserve or remove the seminal vesicle is made on a patient-by-patient basis depending on patient preferences, preoperative and intraoperative findings, and cystic burden.

## 4. Conclusion

We present a rare case of Zinner syndrome with an ectopic ureter, originating from an atrophic kidney, inserted into the ipsilateral seminal vesicle causing recurrent epididymitis. We describe the safe and effective use of robotic surgical techniques to dissect the affected ureter and kidney while sparing the seminal vesicle. Although urologic anatomic abnormalities are rarely diagnosed in adulthood, prompt workup with imaging is recommended for patients who present with recurrent genitourinary symptoms, as it may reveal correctable pathology.

## Data Availability

All relevant data related to this case is included in the report herein. Additional data can be provided upon reasonable request.
